# Risk factors associated with inadequate control of disease activity in elderly patients with rheumatoid arthritis: Results from a nationwide KOrean College of Rheumatology BIOlogics (KOBIO) registry

**DOI:** 10.1371/journal.pone.0205651

**Published:** 2018-10-16

**Authors:** Seung Min Jung, Seung-Ki Kwok, Ji Hyeon Ju, Sang-Won Lee, Jason Jungsik Song, Chong-Hyeon Yoon, Yong-Beom Park, Sung-Hwan Park

**Affiliations:** 1 Division of Rheumatology, Department of Internal Medicine, Yonsei University College of Medicine, Seoul, Korea; 2 Division of Rheumatology, Department of Internal Medicine, College of Medicine, The Catholic University of Korea, Seoul, Republic of Korea; Campus Bio-Medico University of Roma, ITALY

## Abstract

**Objective:**

The proportion of elderly patients with rheumatoid arthritis (RA) is continuously growing as a result of the increasing aging population. We compared disease activity between different age groups, and evaluated the clinical factors associated with high disease activity.

**Methods:**

This cross-sectional study analyzed the data of RA patients enrolled in the Korean College of Rheumatology Biologics registry (KOBIO-RA) between 2012 and 2014. Disease activity between elderly (age ≥ 65 years) and non-elderly patients (age < 65 years) was compared, and the association of clinical factors with high disease activity was assessed using a multivariate logistic regression model.

**Results:**

Of 1,227 patients in KOBIO-RA, 244 patients with RA were aged 65 years or over. In elderly patients, the proportion of men was higher (*P* = 0.012), and the duration of disease was longer (*P* < 0.001) compared with non-elderly patients. The elderly group showed a higher incidence of comorbidity (*P* < 0.001), and less use of methotrexate (*P* = 0.004). Assessment of disease activity using various composite measures showed a higher proportion of high disease activity in elderly patients than non-elderly patients. Longer disease duration, presence of comorbidity, and non-use of methotrexate were independently associated with high disease activity (*P* = 0.002, *P* < 0.001, and *P* = 0.029, respectively).

**Conclusions:**

At enrollment of KOBIO-RA, elderly patients showed higher disease activity compared with non-elderly patients. Disease duration, use of methotrexate, and comorbidity are associated with disease activity control in Korean patients with RA.

## Introduction

Rheumatoid arthritis (RA) is a chronic inflammatory disease of unknown etiology, characterized by peripheral polyarthritis. Approximately 1% of the general population worldwide is affected by RA, with an increasing prevalence in the older population [1]. As the aging population continues to grow, the mean age of RA patients in clinical studies has continuously increased for several decades. In Korea, the population aged 65 years and over is expected to account for 25% of the general population by 2030 [2]. Among patients with RA, the proportion of elderly patients is rapidly growing.

Because age is one of the major determinants in clinical decision-making, there are distinct considerations for treatment strategies and clinical outcomes for elderly patients with RA. Elderly patients frequently have other medical conditions that restrict tight control of RA activity. The current consensus on RA treatment emphasizes treat-to-target strategy for better clinical outcomes [3]. The therapeutic target is disease remission, and if this is unattainable, then the target is maintaining a low disease activity. However, it is difficult to determine the optimal treatment in elderly patients with comorbidities. Although disease-modifying anti-rheumatic drugs (DMARDs) are beneficial to reach the target, elderly patients are at increased risk of developing side effects of DMARDs owing to organ dysfunction. Moreover, immune system aging makes elderly patients more susceptible to infection during immunosuppressive therapy [4]. Thus, physicians often hesitate to use intensive treatment for elderly patients with RA.

Currently, there has been increasing attention on elderly patients with RA among rheumatologists. Recent studies have demonstrated different features in patients that developed RA at an older age. Elderly onset RA has a higher proportion of male patients, and it develops more abruptly [5–7]. In terms of clinical outcomes, patients with elderly onset RA tend to have severe functional disabilities and poor life quality compared with younger onset RA patients [5,8,9]. However, there are still limited data concerning the clinical features and outcomes of elderly patients with RA. As previous studies have mainly focused on the onset age of RA rather than the present age of patients, it is difficult to identify the practical impact of age on disease control. Furthermore, most clinical trials exclude patients with risk factors such as old age and comorbid disease for safety issues. To understand the characteristics of elderly patients with RA in real clinical practice, the clinical profiles and outcome measures in elderly patients should be reviewed using a large patient registry.

In this study, we investigated the clinical features and disease control status in elderly patients with RA using a nationwide registry. We also analyzed the clinical factors affecting insufficient control of RA activity.

## Methods

### Patient population

The Korean College of Rheumatology Biologics Registry for RA (KOBIO-RA) is a nationwide, multi-center cohort that aims to evaluate the clinical outcomes and adverse effects of treatment with biologics in Korean patients with RA. Patients with RA were recruited from 38 hospitals in South Korea since 2012. The enrolled patients were evaluated by rheumatologists, and an informed consent was obtained from all subjects.

The registry consists of two arms: one arm comprises patients starting or switching to a new biologic agent (biologics group), and the other arm comprises patients treated with conventional synthetic DMARDs (control group). Biologic agents comprised rituximab, abatacept, tocilizumab, and tumor necrosis factor inhibitors, including etanercept, infliximab, adalimumab, golimumab, and certolizumab. If a patient in the control group began biologic agent therapy, this patient was reclassified into the biologics group. In Korea, the health care reimbursement system permits biologic agent use in RA patients who have an inadequate response to more than one conventional DMARDs. Further, it is recommended that patients are prescribed with one DMARD, mainly methotrexate (MTX), concurrently with a biologic agent.

The present study is based on cross-sectional data of patient characteristics at enrollment in KOBIO-RA between 2012 and 2014. For comparison between non-elderly and elderly patients, all patients were classified by age at enrollment. Patients aged 65 years or over were categorized as elderly patients, and others were considered non-elderly patients. Names and hospital identification numbers were removed to ensure patient confidentiality. Ethical approval of KOBIO-RA was obtained by all of the institutional review boards of 38 participating institutions, including Yonsei University College of Medicine (4-2013-0075) and the Catholic University College of Medicine (KC12OIMI0675).

### Clinical profiles

An electrical case report form was employed via the Internet to gather patient data [10]. Data included demographics, previous or current use of medications, comorbidities, and extra-articular manifestations. The clinical information was mainly obtained from health questionnaires and interviews with patients.

The investigators of each institution collected laboratory and radiologic findings. Laboratory tests included complete blood count, blood chemistry, and autoantibodies, such as rheumatoid factor and anti-cyclic citrullinated peptide antibody (ACPA). Structural damage, including bony erosion and joint space narrowing, was evaluated on plain radiographs of the hands and feet.

To guarantee the quality of the data, the investigators participated in a workshop supervised by the central task force of the Korean College of Rheumatology. Evaluation was performed at inclusion, initiation, or change of biologic agent, and at every follow-up.

### Comorbidities

To evaluate the clinical impact of comorbidities, the presence of comorbid disease was assessed in the entire study population. Patients were asked about previous diagnoses of other diseases. Additionally, accessible medical records or medications lists were verified by the investigators at each institution. To detect pulmonary comorbidities, chest imaging and pulmonary function tests were reviewed. Chronic obstructive pulmonary disease and restrictive lung disease were determined by abnormalities in pulmonary function tests. Interstitial lung disease, combined with RA, was confined to relevant findings in imaging studies. Chronic kidney disease was defined as estimated glomerular filtration rate less than 60 ml/min calculated using the Modification of Diet in Renal Disease Study equation. Comorbidity information was summarized into numeric scores using the Charlson Comorbidity Index (CCI) [11] and the Elixhauser’s Comorbidity Measure (ECM) [12] to compare the effect of multiple comorbid conditions.

### Assessment of disease activity

The disease activity of all patients was evaluated using validated composite measures at every evaluation. Trained investigators at each institution performed joint assessments. Disease activity was presented with various indices, including disease activity score in 28 joints (DAS28) using erythrocyte sedimentation rate (ESR) or C-reactive protein (CRP) (DAS28-ESR and DAS28-CRP, respectively), simplified disease activity index (SDAI), and clinical disease activity index (CDAI). Based on the American College of Rheumatology-European League against Rheumatism criteria, the current disease status was categorized into remission, or high, moderate, or low disease activity [13].

The functional capacity of patients with RA was determined using a specialized tool, the Routine Assessment of Patient Index Data 3 (RAPID3) [14]. All patients filled in the questionnaire at every data collection point.

### Statistical analysis

Characteristics of non-elderly and elderly patients in KOBIO-RA were compared using Student’s *t*-test and the chi-square test. A multivariate logistic regression model was used to determine the independent risk factors for high disease activity of RA. The clinical variables with *P* ≤ 0.10 in univariate analysis were included in the multivariate regression model. *P*-values <0.05 were considered significant. All statistical analyses were performed using SAS 9.1 software (SAS Institute, Inc., Cary, NC, USA).

## Results

### Patient characteristics

The baseline characteristics of patients in KOBIO-RA are presented in Table 1. Patients had mean age of 54.2 years, and 86.1% of patients were women. Of 1,227 patients at inclusion, 244 patients were classified as elderly patients (age ≥ 65 years). The proportion of men was higher and disease duration was longer in elderly patients compared to non-elderly patients (*P* = 0.012 and *P* < 0.001, respectively). Seropositivity for rheumatoid factor and ACPA, and the presence of radiographic erosion was comparable between non-elderly and elderly patients with RA. Extra-articular manifestations of RA were also comparable in both groups, although interstitial lung disease was more common in elderly patients (*P* = 0.025).

**Table 1:** Baseline characteristics of RA patients at enrollment of KOBIO-RA.

		Total (n = 1227)	Non-elderly patients (age < 65 years) (n = 983)	Elderly patients (age ≥ 65 years) (n = 244)	*P*-value
Demographics	Age (years), mean ± SD	54.2 ± 12.4	50.2 ± 10.4	70.3 ± 4.2	<0.001
	Sex (women), n (%)	1057 (86.1)	859 (87.4)	198 (81.1)	0.012
	Duration of education (years), mean ± SD	11.4 ± 3.7	12.1 ± 3.3	8.8 ± 4.0	<0.001
	Smoking				0.468
	Current smoker, n (%)	91 (7.4)	73 (7.4)	18 (7.4)	
	Ex-smoker, n (%)	85 (6.9)	63 (6.4)	22 (9.0)	
RA-related features	Age at RA diagnosis, mean ± SD	45.4 ± 14.5	42.1 ± 12.7	58.4 ± 14.2	<0.001
	Duration of disease (years), mean ± SD	7.9 ± 7.4	7.4 ± 7.0	9.9 ± 8.7	<0.001
	Seropositivity, n (%)	1095/1173 (93.4)	877 (93.2)	218 (94.0)	0.675
	Rheumatoid factor-positive, n (%)	1001/1190 (84.1)	794 (83.3)	207 (87.3)	0.129
	ACPA-positive, n (%)	844/1003 (84.1)	687 (84.0)	157 (84.9)	0.767
	Erosive arthritis, n (%)	481/870 (55.3)	390 (54.9)	91 (57.2)	0.585
Extra-articular	Rheumatoid nodule, n (%)	25 (2.1)	17 (1.7)	8 (3.4)	0.162
manifestations	Interstitial lung disease, n (%)	19 (1.6)	11 (1.1)	8 (3.4)	0.025
	Pleuritis, n (%)	4 (0.3)	3 (0.3)	1 (0.4)	0.538
	Glomerulonephritis, n (%)	3 (0.2)	1 (0.1)	2 (0.8)	0.068
	Scleritis, n (%)	2 (0.2)	1 (0.1)	1 (0.4)	0.311
	Cutaneous vasculitis, n (%)	2 (0.2)	2 (0.2)	0 (0.0)	0.437
	Secondary Sjögren’s syndrome, n (%)	28 (2.3)	20 (2.1)	8 (3.4)	0.270
Comorbidities	Hypertension, n (%)	318 (25.9)	198 (20.1)	120 (49.2)	<0.001
	Diabetes, n (%)	104 (8.5)	50 (5.1)	54 (22.1)	<0.001
	Cerebrovascular disease, n (%)	9 (0.7)	3 (0.3)	6 (2.5)	<0.001
	Ischemic heart disease, n (%)	17 (1.4)	7 (0.7)	10 (4.1)	<0.001
	Congestive heart failure, n (%)	3 (0.2)	0 (0.0)	3 (1.2)	<0.001
	Peripheral vascular disease, n (%)	3 (0.2)	2 (0.2)	1 (0.4)	0.559
	COPD, n (%)	15 (1.2)	6 (0.6)	9 (3.7)	<0.001
	Restrictive lung disease, n (%)	22 (1.8)	12 (1.2)	10 (4.1)	0.002
	Chronic kidney disease, n (%)	51 (4.2)	20 (2.0)	31 (12.7)	<0.001
	Liver disease, n (%)	47 (3.8)	42 (4.3)	5 (2.0)	0.105
	Peptic ulcer disease, n (%)	48 (3.9)	31 (3.2)	17 (7.0)	0.006
	Depression, n (%)	26 (2.1)	21 (2.1)	5 (2.0)	0.933
	Hematologic malignancies, n (%)	2 (0.2)	2 (0.2)	0 (0.0)	0.481
	Solid tumor, n (%)	13 (1.1)	9 (0.9)	4 (1.6)	0.323
	Metastatic tumor, n (%)	1 (0.1)	1 (0.1)	0 (0.0)	0.618
	Charlson Comorbidity Index, mean ± SD	1.39 ± 0.95	1.26 ± 0.79	1.88 ± 1.33	<0.001
	Elixhauser’s Comorbidity Measure, mean ± SD	1.83 ± 0.94	1.70 ± 0.85	2.38 ± 1.06	<0.001
Medications	Synthetic DMARDs				
	Methotrexate, n (%)	1043 (85.0)	852 (86.7)	191 (78.3)	0.004
	Leflunomide, n (%)	318 (25.9)	248 (25.2)	70 (28.7)	0.397
	Sulfasalazine, n (%)	143 (11.7)	112 (11.4)	31 (12.7)	0.649
	Hydroxychloroquine, n (%)	318 (25.9)	260 (26.4)	58 (23.8)	0.563
	Tacrolimus, n (%)	149 (12.1)	117 (11.9)	32 (13.1)	0.668
	Dual DMARD therapy, n (%)	585 (48.4)	466 (48.1)	119 (49.8)	0.638
	Triple DMARD therapy, n (%)	94 (7.7)	79 (8.0)	15 (6.1)	0.487
	Quadruple DMARD therapy, n (%)	8 (0.7)	6 (0.6)	2 (0.8)	0.727
	Corticosteroids, n (%)	971 (79.1)	780 (79.3)	191 (78.3)	0.713
	Dose of corticosteroids (mg/day), mean ± SD	4.1 ± 3.8	3.9 ± 3.3	4.1 ± 3.9	0.357
	Biologic agents, n (%)	200 (16.3)	162 (16.5)	38 (15.6)	0.732
	Tumor necrosis factor inhibitors, n (%)	170 (13.9)	136 (13.8)	34 (13.9)	0.968
	Rituximab, n (%)	10 (0.8)	9 (0.9)	1 (0.4)	0.432
	Abatacept, n (%)	15 (1.2)	12 (1.2)	3 (1.2)	0.991
	Tocilizumab, n (%)	5 (0.4)	5 (0.5)	0 (0.0)	0.264

SD, standard deviation; RA, rheumatoid arthritis; ACPA, anti-cyclic citrullinated peptide antibody; COPD, chronic obstructive pulmonary disease; DMARD, disease modifying anti-rheumatic drug.

The two groups had a significant difference in comorbid conditions, including hypertension, diabetes, cardiovascular disease, pulmonary disease, renal insufficiency, and peptic ulcer. The prevalence of these diseases was higher in elderly patients, as expected. Accordingly, comorbidity indices (CCI and ECM) were elevated in the elderly group. Comparison of current medications revealed a less common use of MTX in elderly patients (78.3% in the elderly group and 86.5% in the non-elderly group, *P* = 0.004). Other medications, including synthetic conventional DMARDs, corticosteroids, and biologic agents, were similarly prescribed in both groups. The frequency of combination therapy with more than one DMARD was not significantly different between non-elderly and elderly patients.

### Disease activity

As shown in Table 2, disease activity in all included patients was evaluated with various measures at inclusion. Among acute phase reactants, ESR was higher in elderly patients. However, the CRP level was not significantly different between the two groups.

**Table 2:** Composite measures of disease activity in KOBIO-RA patients.

		Total (n = 1227)	Non-elderly patients (age < 65 years) (n = 983)	Elderly patients (age ≥ 65 years) (n = 244)	*P*-value
Disease activity	ESR (mm/hr), mean ± SEM	41.9 ± 0.8	40.6 ± 0.9	47.2 ± 2.0	0.002
	CRP (mg/dl), mean ± SEM	1.70 ± 0.08	1.66 ± 0.09	1.88 ± 0.16	0.231
	Swollen joint count 28, mean ± SEM	5.0 ± 0.2	5.0 ± 0.2	5.4 ± 0.4	0.378
	Tender joint count 28, mean ± SEM	6.7 ± 0.2	6.4 ± 0.2	7.9 ±0.6	0.026
	Patient global assessment, mean ± SEM	5.3 ± 0.1	5.2 ± 0.1	5.6 ± 0.2	0.047
	Physician global assessment, mean ± SEM	4.6 ± 0.1	4.6 ± 0.1	4.6 ± 0.2	0.994
	DAS28-ESR, mean ± SEM	4.64 ± 0.05	4.58 ± 0.05	4.89 ± 0.12	0.013
	DAS28-CRP, mean ± SEM	3.95 ± 0.05	3.90 ± 0.05	4.16 ± 0.12	0.045
	SDAI, mean ± SEM	21.40 ± 0.44	20.82 ± 0.47	23.75 ± 1.09	0.014
	CDAI, mean ± SEM	19.6 ± 0.4	19.2 ± 0.4	21.5 ± 1.0	0.036
	State of disease activity based on DAS28-CRP				0.035
	Remission, n (%)	338 (27.5)	273 (27.8)	65 (26.6)	
	Low disease activity, n (%)	112 (9.1)	91 (9.3)	21 (8.6)	
	Moderate disease activity, n (%)	445 (36.3)	371 (37.7)	74 (30.3)	
	High disease activity, n (%)	315 (25.7)	237 (24.1)	78 (32.0)	
Function	RAPID3, mean ± SD	12.31 ± 6.82	12.00 ± 6.72	13.55 ± 7.09	0.002

SEM, standard error of mean; ESR, erythrocyte sedimentation rate; CRP, C-reactive protein; DAS-28, disease activity score in 28 joints; SDAI, simplified disease activity index; CDAI, clinical disease activity index; RAPID3, routine assessment of patient index data 3.

Elderly patients had more tender joints but comparably swollen joints to non-elderly patients. There was a discrepancy in the perception of disease activity by patients and physicians, which was worse according to the patients’ assessment. Although the global assessment by physicians did not differ significantly in both groups, elderly patients perceived their disease more severely than non-elderly patients.

**Fig 1:**
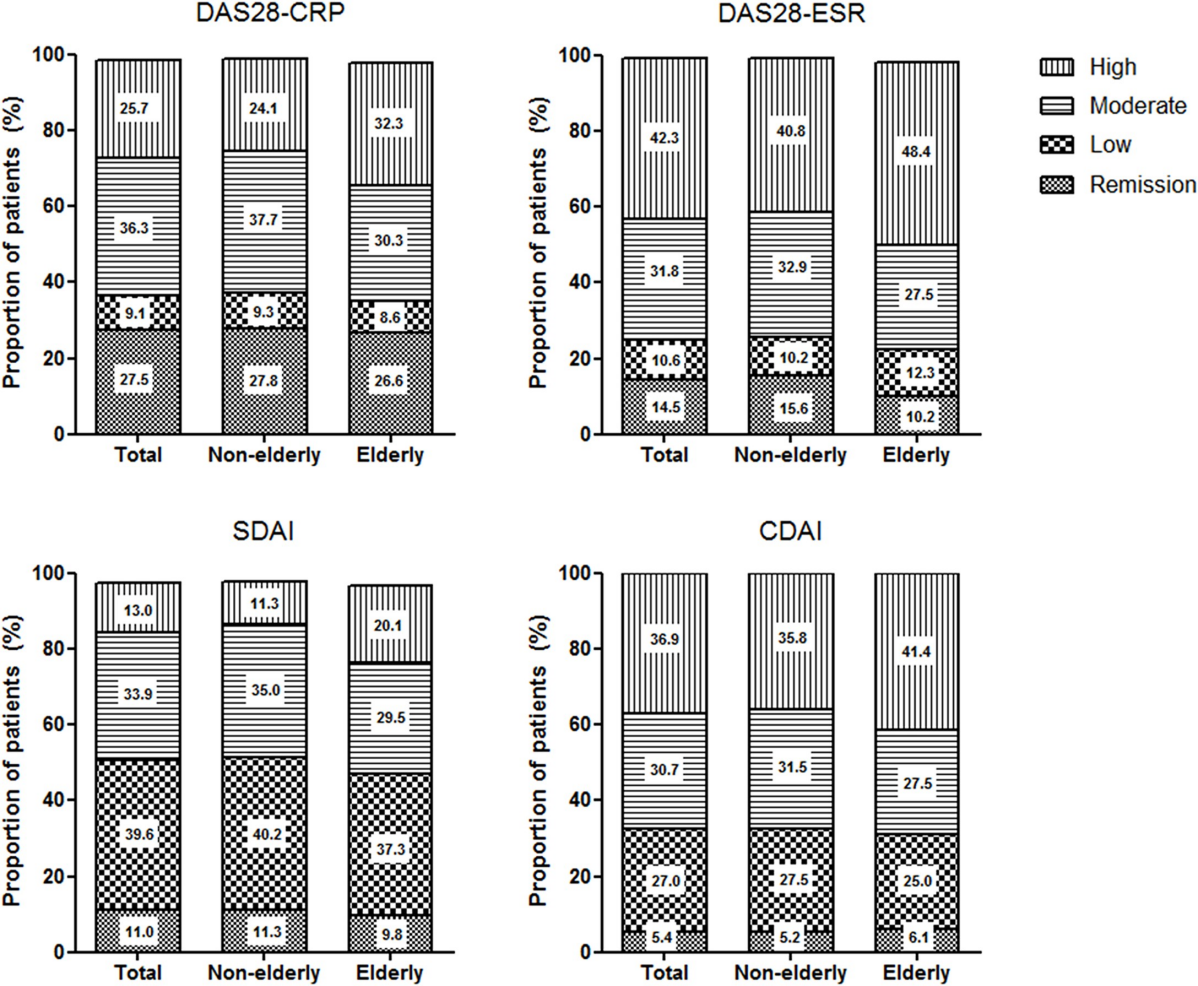
Classification of disease activity in KOBIO-RA patients. Current rheumatoid arthritis (RA) activity in KOBIO-RA was determined by multiple validated composite measures of disease activity including joint counts: disease activity score in 28 joints using C-reactive protein (DAS28-CRP) or erythrocyte sedimentation rate (DAS28-ESR); simplified disease activity index (SDAI); and clinical disease activity index (CDAI). According to the American College of Rheumatology-European League against Rheumatism criteria, disease activity was classified into remission or low, moderate, or high disease activity.

The evaluation of the disease activity based on clinical and laboratory parameters revealed a higher disease activity in elderly patients compared to non-elderly patients. The scores of all activity indices were significantly higher in the elderly group. Most of the included patients (62.0%) had moderate to high disease activity despite treatment because the biologics group of KOBIO-RA consisted of patients with an inadequate response to conventional DMARDs or prior biologics. Of note, more patients in the elderly group were classified as having high disease activity compared to the non-elderly group (24.1% *vs* 32.0% according to DAS28-CRP) (Fig 1). Functional impairment and pain evaluated by RAPID 3 were more severe in elderly patients, and this finding was consistent with the result that elderly patients showed higher disease activity.

### Risk factors for high disease activity

**Fig 2:**
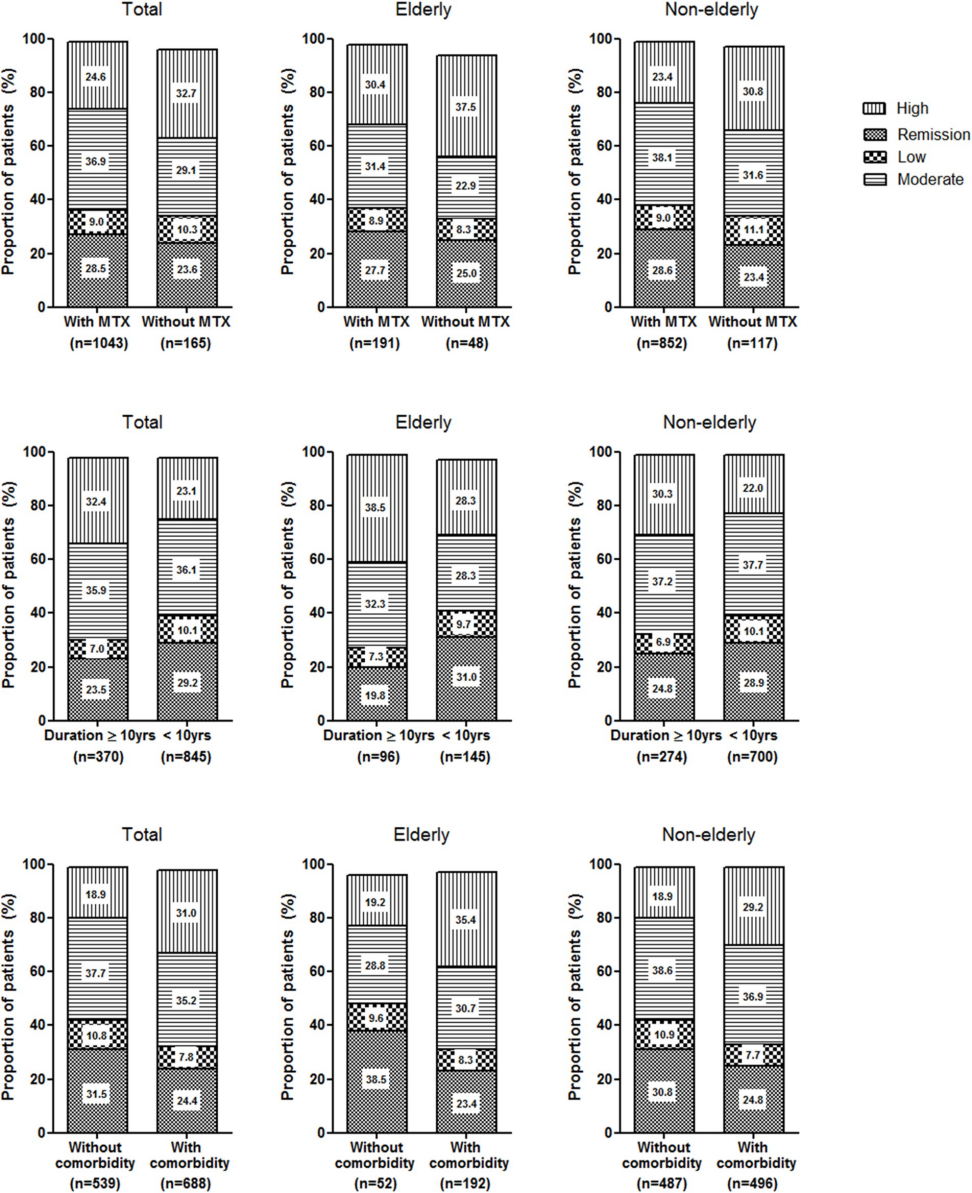
Assessment of disease activity according to the risk factors. Disease activity in the presence or absence of risk factors (**A**. non-use of MTX; **B**. disease duration over 10 years; **C**. presence of comorbidity) was evaluated using DAS28-CRP in elderly and non-elderly patients, respectively. The distribution of remission, and low, moderate, and high disease activity showed a similar pattern in the entire study population, the elderly group, and the non-elderly group.

Because the difference in disease activity of elderly and non-elderly patients was primarily high disease activity, the association between clinical profiles and high disease activity was investigated (Table 3). As ESR is influenced by age, disease activity was classified based on DAS28-CRP. For the entire study population, old age, elderly onset of RA, and longer disease duration were related to high disease activity in univariate analysis. The disease activity of RA was also affected by education level, comorbidity, and MTX use. Male sex and presence of interstitial lung disease had no significant impact on disease activity. Multivariate analysis of significant factors revealed that longstanding RA over 10 years, presence of comorbid disease, and no use of MTX were independent risk factors for high disease activity in KOBIO-RA. When the disease activity was separately evaluated in elderly and non-elderly patients, these risk factors affected disease activity in a similar pattern (Fig 2). The comorbidity indices had linear correlations with DAS28-ESR and DAS28-CRP (all *P* < 0.001).

**Table 3:** Risk factors for high disease activity in KOBIO-RA patients.

	Univariate		Multivariate	
	OR (95% CI)	*P*-value	OR (95% CI)	*P*-value
Old age (≥ 65 years)	1.512 (1.111–2.057)	0.008		
Male	1.046 (0.724–1.512)	0.811		
Elderly onset of RA (≥ 60 years)	1.479 (1.056–2.073)	0.023		
Longer duration of disease (≥ 10 years)	1.593 (1.215–2.089)	0.001	1.618 (1.201–2.180)	0.002
Lower education level (≤ 12 years)	1.356 (1.011–1.819)	0.042		
Presence of ILD	1.017 (0.363–2.847)	0.974		
Presence of comorbid conditions (ECM ≥ 2 points)	1.940 (1.481–2.541)	<0.001	1.722 (1.297–2.285)	<0.001
No use of methotrexate	1.568 (1.096–2.242)	0.014	1.503 (1.042–2.170)	0.029

OR, odds ratio; RA, rheumatoid arthritis; ILD, interstitial lung disease; ECM, Elixhauser’s Comorbidity Measure; CI, confidence interval.

Because comorbidities were closely associated with high disease activity of RA, the impact of each comorbid disease was analyzed. No comorbidities, except diabetes, showed any significant association with high disease activity in KOBIO-RA. Diabetes had a marginal effect on the disease activity of RA, after adjustment with other covariates (Table 4).

**Table 4:** Comorbid conditions associated with high disease activity in KOBIO-RA patients.

	Univariate		After adjustment*	
	OR (95% CI)	*P*-value	OR (95% CI)	*P*-value
Hypertension	0.940 (0.700–1.263)	0.682		
Diabetes	1.752 (1.148–2.673)	0.009	1.549 (0.997–2.409)	0.052
Cerebrovascular disease	0.353 (0.044–2.834)	0.327		
Ischemic heart disease	0.946 (0.303–2.956)	0.924		
Cardiac arrhythmia	0.947 (0.190–4.715)	0.947		
Hypothyroidism	1.035 (0.527–2.029)	0.921		
COPD	1.912 (0.675–5.414)	0.223		
Restrictive lung disease	1.640 (0.681–3.947)	0.270		
Chronic kidney disease	0.918 (0.473–1.784)	0.918		
Liver disease	1.394 (0.742–2.617)	0.302		
Peptic ulcer disease	1.491 (0.804–2.766)	0.205		

*Adjusted for age, sex, disease duration, and methotrexate treatment.

OR, odds ratio; COPD, chronic obstructive pulmonary disease; CI, confidence interval.

## Discussion

In the present study, elderly RA patients showed higher disease activity at enrollment of KOBIO-RA, compared to non-elderly patients. Various composite measures showed a higher proportion of high disease activity in elderly patients. However, age was not an independent risk factor for high disease activity of RA in entire study population. Patients with high disease activity had longstanding RA and were less frequently treated with MTX. Comorbidities were more common in elderly patients, and the presence of comorbidities was closely linked with high disease activity in RA.

Earlier research has shown that elderly patients with RA undergo less aggressive treatment. Age affected the treatment pattern, especially the use of conventional and biologic DMARDs [15–18]. In contrast, another study suggested similar use of DMARD therapy [19]. In the present study, the use of biologic agents or combination DMARD therapy was not significantly different between elderly and non-elderly patients with RA. The prescription of medications was comparable in both groups, except for MTX. All current guidelines recommend MTX as an anchor drug that should be started as soon as possible after diagnosing RA [20–22]. Treatment with MTX is known to significantly improve clinical outcomes in RA patients. However, the use of MTX is limited in patients with impaired kidney, lung, and liver function. Patients at risk of severe infection also have difficulties in taking MTX. Physicians tend to be reluctant to prescribe MTX for elderly patients with comorbidities and immunosenescence, resulting in unfavorable outcomes in elderly patients with RA [15,16].

In line with these findings, comorbidity was an independent risk factor for uncontrolled disease activity in the whole study population. Comorbidity was previously known to have an independent association with clinical measures, such as the CDAI and the Health Assessment Questionnaire [23,24]. We also found that the presence of comorbid disease has a significant correlation with disease activity indices, including DAS28. The safety concerns and contraindications for DMARDs in patients with comorbidities restrict tight control of disease activity. Moreover, earlier studies suggested decreased adherence to medications in RA patients with comorbid disease [25]. Diabetes was associated with non-adherence to treatment [26], supporting the current observation that patients with diabetes tend to have active RA. Because comorbid conditions affect both patients and health providers in the management of RA, adjustment of outcome measures by comorbidity would be required [27].

Although this study showed higher disease activity in elderly patients, the evaluation of disease activity of elderly patients with RA should be interpreted carefully. Importantly, the difference between elderly and non-elderly patients was mainly determined by subjective measures, rather than objective measures. Elderly patients with RA had worse outcomes in tender joint counts and patient global assessment compared to non-elderly patients, whereas swollen joint counts and physician global assessment were comparable. In general, the aging population is more susceptible to pain measured by the visual analogue scale and global assessment [28]. Additionally, ESR, an important acute phase reactant, is influenced by age, as well as inflammation [29]. To compensate for these limitations, we used various validated composite measures of disease activity that include joint assessment or not. All measures showed a similar pattern that revealed high disease activity in elderly patients with RA.

Interestingly, the proportion of remission and low disease activity were not significantly different between elderly and non-elderly patients. Although the primary target for treatment of RA is to reach a state of clinical remission, other treatment goal than remission should be chosen according to presence of comorbidity, and patient factors such as aging, with consideration of the safety aspect [3]. The therapeutic target can be generally adapted to reach remission or low disease activity. Based on this general principle, treatment goal attainment was comparable in elderly and non-elderly patients. However, structural destruction and functional disability can progress in proportion to disease activity. Thus, RA activity should be maintained as low as possible, even though the therapeutic goal is not reached.

The present study has several limitations. First, there was a selection bias affecting the disease activity of the entire study population. The biologics group, accounting for more than half of patients enrolled in KOBIO-RA, was comprised of patients with an inadequate response to prior treatment. At enrollment, these patients had moderate to high disease activity. Thus, the patients in KOBIO-RA had more active RA compared to all patients with RA. Actually, the mean DAS28 and the proportion of patients with moderate to high disease activity were higher in KOBIO-RA compared to other RA registries in Korea [30,31]. Secondly, this study was based on cross-sectional data, and the response to RA treatment was not evaluated. Additional analysis from longitudinal data is required to investigate treatment outcomes in elderly patients with RA. Nevertheless, KOBIO-RA provides a better understanding of the nationwide characteristics of patients with RA in Korea.

In conclusion, elderly patients with RA had higher disease activity compared with non-elderly patients at enrollment of Korean nationwide biologics registry. High disease activity was associated with longer disease duration, limited use of MTX, and presence of comorbidities, rather than age itself. Patient characteristics need to be considered in the treatment of RA, especially in elderly patients at increased risk of high disease activity.

## Supporting information

S1 Table Comparison of baseline characteristics between elderly patients and non-elderly patients at enrollment of KOBIO-RA when divided into elderly and non-elderly patients based on the age of 60. (DOC)

S2 Table Difference in composite measures of disease activity between elderly patients and non-elderly patients in KOBIO-RA when divided into elderly and non-elderly patients based on the age of 60. (DOC)

S3 Table Risk factors for high disease activity in KOBIO-RA patients when the definition of old age as one of risk factors is 60 years old or over. (DOC)

## Abbreviations

RA: rheumatoid arthritis
DMARDs: disease modifying anti-rheumatic drugs
KOBIO-RA: Korean College of Rheumatology Biologics Registry for Rheumatoid Arthritis
MTX: methotrexate
ACPA: anti-cyclic citrullinated peptide antibody
CCI: Charlson Comorbidity Index
ECM: Elixhauser’s Comorbidity Measure
DAS28: disease activity score in 28 joints
ESR: erythrocyte sedimentation rate
CRP: C-reactive protein
SDAI: simplified disease activity index
CDAI: clinical disease activity index (CDAI)
RAPID3: routine assessment of patient index data 3
