# Supplementary material for: Risk factors associated with inadequate control of disease activity in elderly patients with rheumatoid arthritis: Results from a nationwide KOrean College of Rheumatology BIOlogics (KOBIO) registry
Source: PLoS One. 2018 Oct 16;13(10):e0205651. doi: 10.1371/journal.pone.0205651 (PMC6191131; doi:10.1371/journal.pone.0205651)
Supplement: S1 Table — (DOC) [file pone.0205651.s001.doc]

**S1 Table. Baseline characteristics of RA patients at enrollment of KOBIO-RA**

|  |  | Total  (n = 1227) | Non-elderly patients  (age < 60 years)  (n = 797) | Elderly patients  (age ≥ 60 years)  (n = 430) | *P*-value |
| --- | --- | --- | --- | --- | --- |
| Demographics | Age (years), mean ± SD | 54.2 ± 12.4 | 47.5 ± 9.7 | 66.7 ± 5.3 | <0.001 |
|  | Sex (women), n (%) | 1057 (86.1) | 704 (88.3) | 353 (82.1) | 0.003 |
|  | Duration of education (years), mean ± SD | 11.4 ± 3.7 | 12.6 ± 3.1 | 9.3 ± 3.9 | <0.001 |
|  | Smoking |  |  |  | 0.210 |
|  | Current smoker, n (%) | 91 (7.4) | 63 (7.9) | 28 (6.5) |  |
|  | Ex-smoker, n (%) | 85 (6.9) | 48 (6.0) | 37 (8.6) |  |
| RA-related features | Age at RA diagnosis, mean ± SD | 45.4 ± 14.5 | 39.7 ± 12.0 | 55.8 ± 13.1 | <0.001 |
|  | Duration of disease (years), mean ± SD | 7.9 ± 7.4 | 7.1 ± 6.8 | 9.3 ± 8.4 | <0.001 |
|  | Seropositivity, n (%) | 1095/1173 (93.4) | 706 (92.4) | 389 (95.1) | 0.077 |
|  | Rheumatoid factor-positive, n (%) | 1001/1190 (84.1) | 634 (82.0) | 367 (88.0) | 0.007 |
|  | ACPA-positive, n (%) | 844/1003 (84.1) | 556 (83.4) | 288 (85.7) | 0.335 |
|  | Erosive arthritis, n (%) | 481/870 (55.3) | 318 (55.2) | 163 (55.4) | 0.948 |
| Extra-articular | Rheumatoid nodule, n (%) | 25 (2.1) | 13 (1.6) | 12 (2.8) | 0.199 |
| manifestations | Interstitial lung disease, n (%) | 19 (1.6) | 6 (0.8) | 13 (3.0) | 0.025 |
|  | Pleuritis, n (%) | 4 (0.3) | 2 (0.3) | 2 (0.5) | 0.423 |
|  | Glomerulonephritis, n (%) | 3 (0.2) | 1 (0.1) | 2 (0.5) | 0.266 |
|  | Scleritis, n (%) | 2 (0.2) | 1 (0.1) | 1 (0.2) | 0.468 |
|  | Cutaneous vasculitis, n (%) | 2 (0.2) | 2 (0.3) | 0 (0.0) | 0.302 |
|  | Secondary Sjögren’s syndrome, n (%) | 28 (2.3) | 17 (2.1) | 11 (2.6) | 0.459 |
| Comorbidities | Hypertension, n (%) | 318 (25.9) | 119 (14.9) | 199 (46.3) | <0.001 |
|  | Diabetes, n (%) | 104 (8.5) | 33 (4.1) | 71 (16.5) | <0.001 |
|  | Cerebrovascular disease, n (%) | 9 (0.7) | 2 (0.3) | 7 (1.6) | 0.007 |
|  | Ischemic heart disease, n (%) | 17 (1.4) | 4 (0.5) | 13 (3.0) | <0.001 |
|  | Congestive heart failure, n (%) | 3 (0.2) | 0 (0.0) | 3 (0.7) | 0.018 |
|  | Peripheral vascular disease, n (%) | 3 (0.2) | 2 (0.0) | 1 (0.2) | 0.950 |
|  | COPD, n (%) | 15 (1.2) | 5 (0.6) | 10 (2.3) | 0.010 |
|  | Restrictive lung disease, n (%) | 22 (1.8) | 10 (1.3) | 12 (2.8) | 0.053 |
|  | Chronic kidney disease, n (%) | 51 (4.2) | 10 (1.3) | 41 (9.5) | <0.001 |
|  | Liver disease, n (%) | 47 (3.8) | 29 (3.6) | 18 (4.2) | 0.634 |
|  | Peptic ulcer disease, n (%) | 48 (3.9) | 27 (3.4) | 21 (4.9) | 0.197 |
|  | Depression, n (%) | 26 (2.1) | 16 (2.0) | 10 (2.3) | 0.712 |
|  | Hematologic malignancies, n (%) | 2 (0.2) | 2 (0.3) | 0 (0.0) | 0.299 |
|  | Solid tumor, n (%) | 13 (1.1) | 4 (0.5) | 6 (1.4) | 0.097 |
|  | Metastatic tumor, n (%) | 1 (0.1) | 1 (0.1) | 0 (0.0) | 0.462 |
|  | Charlson Comorbidity Index, mean ± SD | 1.39 ± 0.95 | 1.22 ± 0.71 | 1.69 ± 1.23 | <0.001 |
|  | Elixhauser’s Comorbidity Measure, mean ± SD | 1.83 ± 0.94 | 1.61 ± 0.77 | 2.25 ± 1.07 | <0.001 |
| Medications | Synthetic DMARDs |  |  |  |  |
|  | Methotrexate, n (%) | 1043 (85.0) | 691 (88.1) | 352 (83.0) | 0.013 |
|  | Leflunomide, n (%) | 318 (25.9) | 201 (25.6) | 117 (27.6) | 0.461 |
|  | Sulfasalazine, n (%) | 143 (11.7) | 86 (11.0) | 57 (13.4) | 0.204 |
|  | Hydroxychloroquine, n (%) | 318 (25.9) | 206 (26.3) | 112 (26.4) | 0.958 |
|  | Tacrolimus, n (%) | 149 (12.1) | 93 (11.9) | 56 (13.2) | 0.497 |
|  | Dual DMARD therapy, n (%) | 585 (48.4) | 375 (47.8) | 210 (49.5) | 0.573 |
|  | Triple DMARD therapy, n (%) | 94 (7.7) | 62 (7.9) | 32 (7.5) | 0.823 |
|  | Quadruple DMARD therapy, n (%) | 8 (0.7) | 5 (0.6) | 3 (0.7) | 0.886 |
|  | Corticosteroids, n (%) | 971 (79.1) | 635 (79.7) | 336 (78.1) | 0.528 |
|  | Dose of prednisolone (mg/day), mean ± SD | 4.1 ± 3.8 | 4.2 ± 4.0 | 3.9 ± 3.2 | 0.175 |
|  | Biologic agents, n (%) | 200 (16.3) | 162 (16.5) | 38 (15.6) | 0.732 |
|  | Tumor necrosis factor inhibitors, n (%) | 170 (13.9) | 112 (14.1) | 58 (13.5) | 0.785 |
|  | Rituximab, n (%) | 10 (0.8) | 6 (0.8) | 4 (0.9) | 0.742 |
|  | Abatacept, n (%) | 15 (1.2) | 10 (1.3) | 5 (1.2) | 0.889 |
|  | Tocilizumab, n (%) | 5 (0.4) | 5 (0.6) | 0 (0.0) | 0.100 |

SD, standard deviation; RA, rheumatoid arthritis; ACPA, anti-cyclic citrullinated peptide antibody; COPD, chronic obstructive pulmonary disease; DMARD, disease modifying anti-rheumatic drug.
