# Supplementary material for: Risk factors associated with inadequate control of disease activity in elderly patients with rheumatoid arthritis: Results from a nationwide KOrean College of Rheumatology BIOlogics (KOBIO) registry
Source: PLoS One. 2018 Oct 16;13(10):e0205651. doi: 10.1371/journal.pone.0205651 (PMC6191131; doi:10.1371/journal.pone.0205651)
Supplement: S2 Table — (DOC) [file pone.0205651.s002.doc]

**S2 Table. Composite measures of disease activity in KOBIO-RA patients**

|  |  | Total  (n = 1227) | Non-elderly patients  (age < 60 years)  (n = 797) | Elderly  patients  (age ≥ 60 years)  (n = 430) | *P*-value |
| --- | --- | --- | --- | --- | --- |
| Disease activity | ESR (mm/hr), mean ± SEM | 41.9 ± 0.8 | 40.3 ± 1.0 | 45.9 ± 1.4 | 0.001 |
|  | CRP (mg/dl), mean ± SEM | 1.70 ± 0.08 | 1.69 ± 0.11 | 1.79 ± 0.11 | 0.555 |
|  | Swollen joint count 28, mean ± SEM | 5.0 ± 0.2 | 5.0 ± 0.2 | 5.0 ± 0.3 | 0.856 |
|  | Tender joint count 28, mean ± SEM | 6.7 ± 0.2 | 6.4 ± 0.3 | 7.3 ±0.4 | 0.090 |
|  | Patient global assessment, mean ± SEM | 5.3 ± 0.1 | 5.2 ± 0.1 | 5.4 ± 0.1 | 0.098 |
|  | Physician global assessment, mean ± SEM | 4.6 ± 0.1 | 4.6 ± 0.1 | 4.7 ± 0.1 | 0.454 |
|  | DAS28-ESR, mean ± SEM | 4.64 ± 0.05 | 4.55 ± 0.06 | 4.80 ± 0.09 | 0.020 |
|  | DAS28-CRP, mean ± SEM | 3.95 ± 0.05 | 3.89 ± 0.06 | 4.06 ± 0.08 | 0.086 |
|  | SDAI, mean ± SEM | 21.40 ± 0.44 | 20.80 ± 0.53 | 22.53 ± 0.77 | 0.058 |
|  | CDAI, mean ± SEM | 19.6 ± 0.4 | 19.2 ± 0.4 | 20.5 ± 0.7 | 0.092 |
|  | State of disease activity based on DAS28-CRP |  |  |  | 0.004 |
|  | Remission, n (%) | 338 (27.5) | 226 (28.4) | 112 (26.0) |  |
|  | Low disease activity, n (%) | 112 (9.1) | 73 (9.2) | 39 (9.1) |  |
|  | Moderate disease activity, n (%) | 445 (36.3) | 304 (38.1) | 141 (32.8) |  |
|  | High disease activity, n (%) | 315 (25.7) | 189 (23.7) | 126 (29.3) |  |
| Function | RAPID3, mean ± SD | 12.31 ± 6.82 | 11.89 ± 6.71 | 13.09 ± 6.97 | 0.003 |

SEM, standard error of mean; ESR, erythrocyte sedimentation rate; CRP, C-reactive protein; DAS-28, disease activity score in 28 joints; SDAI, simplified disease activity index; CDAI, clinical disease activity index; RAPID3, routine assessment of patient index data 3.
