# Supplementary material for: Risk factors associated with inadequate control of disease activity in elderly patients with rheumatoid arthritis: Results from a nationwide KOrean College of Rheumatology BIOlogics (KOBIO) registry
Source: PLoS One. 2018 Oct 16;13(10):e0205651. doi: 10.1371/journal.pone.0205651 (PMC6191131; doi:10.1371/journal.pone.0205651)
Supplement: S3 Table — (DOC) [file pone.0205651.s003.doc]

S3 Table. Risk factors for high disease activity in KOBIO-RA patients

|  | Univariate |  | Multivariate |  |
| --- | --- | --- | --- | --- |
|  | OR (95% CI) | *P*-value | OR (95% CI) | *P*-value |
| Old age (≥ 60 years) | 1.333 (1.024 – 1.736) | 0.033 |  |  |
| Male | 1.046 (0.724 – 1.512) | 0.811 |  |  |
| Elderly onset of RA (≥ 60 years) | 1.479 (1.056 – 2.073) | 0.023 | 1.643 (1.039 – 2.598) | 0.034 |
| Longer duration of disease (≥ 10 years) | 1.593 (1.215 – 2.089) | 0.001 | 1.654 (1.228 – 2.27) | 0.002 |
| Lower education level (≤ 12 years) | 1.356 (1.011 – 1.819) | 0.042 |  |  |
| Presence of ILD | 1.017 (0.363 – 2.847) | 0.974 |  |  |
| Presence of comorbid conditions (comorbidity index ≥ 2 points) | 1.940 (1.481 – 2.541) | <0.001 | 1.743 (1.313 – 2.313) | <0.001 |
| No use of methotrexate | 1.568 (1.096 – 2.242) | 0.014 | 1.448 (1.007 – 2.081) | 0.046 |

OR, odds ratio; RA, rheumatoid arthritis; ILD, interstitial lung disease; CI, confidence interval.
